# Analysis of Transcript Expression and Core Promoter DNA Sequences of Brain, Adipose Tissues and Testis in Human and Fruit Fly

**DOI:** 10.3390/ijms262211114

**Published:** 2025-11-17

**Authors:** Viktor Vedelek, Peter Juma Ochieng, Anna Vágvölgyi, Olga Nagy, János Zádori, Rita Sinka

**Affiliations:** 1 Department of Genetics, Faculty of Science and Informatics, University of Szeged, 6726 Szeged, Hungary; 2 Center of Reproductive Medicine, Department of Obstetrics and Gynecology, Albert Szent-Györgyi Medical School, University of Szeged, 6723 Szeged, Hungary; 3 Interdisciplinary Research Development and Innovation Center of Excellence, University of Szeged, 6720 Szeged, Hungary; 4 Institute of Informatics, University of Szeged, 6720 Szeged, Hungary; 5 Department of Medicine, Albert Szent-Györgyi Medical School, University of Szeged, 6725 Szeged, Hungary

**Keywords:** transcriptional start site, tissue specificity, ortholog interaction network, brain, adipose tissue, testis

## Abstract

Gene expression plays a fundamental role in defining the characteristics of living organisms. To deepen our understanding of tissue-specific gene expression, we analyzed transcript variant enrichment across different tissues in human and *Drosophila melanogaster*. Datasets are widely accessible for both of these organisms. Given the substantial volume of available information, we have focused our interest on three fundamentally distinct tissues: the brain, where both neuronal and glial cells exhibit a relatively high cellular surface area, thus requiring a large amount of lipids; the adipose tissue, which is well-known for lipid storage; and the testis, which contains a massive number of developing spermatids with high membrane requirement. These three organs have fundamental differences in their structure and function yet share some common features; they all have lipid-rich cells and have special metabolic pathways. Most studies focus on gene expression, and transcript level analyses are less common; therefore, we aimed to characterize the transcript profiles of these tissues and examine evolutionarily conserved pathways between humans and Drosophila. Additionally, we analyzed the flanking sequences of transcriptional start sites of tissue-enriched transcripts. Our findings suggest that Drosophila tissues exhibit more distinct regulation of gene expression in individual tissues (weaker correlation in expression and variable nucleotide content in core promoter), whereas human gene expression is more generalized, likely relying more heavily on distal regulatory elements for tissue-specific expression. Through network analysis, summarizing tissue specificity, physical interactions, and orthologue data, we identified shared central pathways among these tissues. A relatively large network was observable in the testis, where the ubiquitin proteasome system, various kinases and transcription factors showed central position in both organisms. Additionally, we highlighted the evolutionary potential of highly enriched testis-specific transcripts. This work provides valuable insights into the mechanisms underlying tissue-specific gene expression and evolutionary conservation.

## 1. Introduction

The regulation of gene expression is fundamental for the proper function of biological entities. As transcriptomics has advanced, disparities in gene expression among different tissues have become apparent, leading to the identification of specific expression patterns that are characteristic of individual tissues and cells. It is also evident that changes in gene expression can contribute greatly to macro- and micro-evolutionary processes [1].

The brain and testis represent highly specialized organs with considerable similarities (isolation from other tissues, blood barriers, high energy demand, etc.), whereas adipose tissue, which refers to fat in humans and the fat body in Drosophila, is typically less complex. However, the commonality of special lipid metabolism of certain cell types across all three tissues positions them as valuable candidates for comparative studies [2,3,4,5,6,7]. In the brain and testis, lipids are required for membranes; meanwhile, in fat cells, lipids are stored in lipid droplets. Analyzing gene expressions in various tissues has uncovered tissue-specific genes. These genes, while generally not as crucial as those that are widely expressed during the early phases of tissue formation, are important for establishing the unique attributes of the tissues [8]. More complex tissues reveal a higher level of tissue-specific gene expression. Among bilaterian species, the brain and testis are noted for having the most tissue-specific genes, in contrast to adipose tissues, which have the fewest [9]. Although adipose tissue contains a small number of specific genes (38), many of these genes play a crucial role in the tissue’s functional characteristics [10]. Despite both the brain and testis exhibiting a high quantity of tissue-specific genes, the evolutionary rates of these organs diverge significantly. The brain is considered a slowly evolving organ, whereas the testis undergoes evolutionary changes at a more rapid pace [8]. The gene expression and regulation specific to the brain are intensively investigated, given their possible associations with a range of diseases [11,12,13,14]. To unravel the complexities of brain function, data specific to the brain, particular brain regions, and cell types were produced, demonstrating a notable enrichment of transcripts in the diverse units of the nervous system [12,15,16,17]. Transcriptomic analyses greatly improved our knowledge of brain development and function in Drosophila as well [18,19,20].

The substantial number of testis-specific genes expressed in Drosophila leads to the assumption that the testis serves as an evolutionary testing ground for novel genes [21,22]. In the testis, gene expression is notably permissive, allowing for the expression of a vast majority of the genome. The emergence of a novel gene or a functional mutation that exerts a negative influence on testicular function would compromise its transmission to subsequent generations. On the other hand, neutral or beneficial genetic changes can be transmitted, providing the essential genetic variation that could fuel evolutionary development. While testis-specific genes in Drosophila are rarely deemed essential, they frequently attain novel, specialized functions that are vital for the process of spermatogenesis [22,23,24,25,26,27,28]. In recent years, there has been a growing number of publications that concentrate on the investigation of testis-specific genes in mammals. These studies indicate that some of these genes are essential for the normal progression of spermatogenesis (*Samd4a*, *FER1L5*, *AXDND1*, *IRGC1*, *Spem2*), whereas others are not (*Acrv1*, *Adgrf3*, *Atp8b5*, *Cfap90*, *Cfap276*, *Fbxw5*, *Gm17266*, *Lrrd1*, *Mroh7*, *Nemp1*, *Spata45*, and *Trim36*). These data suggest that a similar evolutionary mechanism to that observed in Drosophila might exist in mammals [29,30,31,32,33,34]. In mammals, several highly conserved genes that are predominantly expressed in the testis have been discovered (*TEX*), and mutations in these genes are associated with infertility [35]. Additionally, there is evidence to suggest that testis-enriched genes could be implicated in various ways in the development of tumors [36,37,38].

The specificity of gene expression in eukaryotic organisms could be influenced by a variety of transcription factors, enhancer and suppressor sequences, and the presence of promoter sequences that facilitate the initiation of transcription. Testis-specific transcription initiation complexes were determined in both mammals and Drosophila [39,40,41,42,43]. The regulatory region required for normal gene expression can vary greatly across different genes, tissues, and developmental stages [44]. Core promoter variants play a role in developmental regulatory networks [45]. The core promoter is a short region adjacent to the transcription start site (TSS); it is sufficient to facilitate the assembly of the RNA polymerase II transcription machinery and initiate the transcription process [44,46,47,48]. Transcription factors that bind in proximity (a few hundred base pairs) to the core promoter can alter its function, and distal enhancers (in thousands of base pairs) provide further regulatory options, enabling specific use of a given gene. Core promoters integrate the regulatory effects of distal enhancer elements, which can alter their low basal activity. The expression of testis-specific genes in Drosophila can be effectively regulated using a comparatively short promoter sequence. For example, in the case of beta-2 tubulin, a 53 bp sequence with a 14 bp regulatory element is sufficient to drive testis-specific gene expression [49]. This observation led us to investigate DNA sequences flanking the TSS of highly enriched transcripts in different tissues. The presence of multiple transcriptional start sites (TSSs) within a single gene can give rise to different types of transcripts, and the transcript variety is further increased by the processes of splicing and tissue-specific splicing. The average transcript count per protein-coding gene in humans is roughly 3.4 (GTEx), markedly higher than the approximately 2.2 (FlyBase) found in Drosophila [50,51]. Nevertheless, it is noteworthy that 80% of the genes have a single predominant transcript [52]. Despite this, genes with higher transcript numbers tend to be more essential, and the transcript variants are important in evaluating human genetic variation [53,54]. A greater diversity of transcripts and transcript variants enhances the potential uses of the gene. The importance of transcript variants is well-documented in many biological processes and pathological conditions [55,56,57]. In Drosophila, the orthologue of the tumor suppressor F-box protein FBW7 is encoded by the *archipelago* (*ago*) gene, of which expression and function differ based on the transcript variants [58,59]. The components of the ubiquitin proteasome system also have tissue-specific variants; the testis-specific variant of the E3 backbone Cul3 is crucial for proper spermatid development [60]. *Centrosomin* (*cnnT*) serves as another example of a tissue-specific transcript variant with a well-characterized testis-enriched isoform [61]. Therefore, in the Drosophila testis, both tissue-specific genes and tissue-specific transcripts are crucial for the process of spermatogenesis.

Our aim in this study was to compare the expression profiles of individual transcripts and investigate the expression patterns of transcripts in the brain, adipose tissue and testis between human and Drosophila. In contrast to most transcriptomic investigations, in this study, the comparison was made mostly on transcript levels. This increases the resolution as transcript variants are handled separately and enables a clear association between transcripts and corresponding sequences. We analyzed and compared data from the Drosophila FlyAtlas2 and the Human GTEx tissue expression atlases. By relying on the tissue-specificity data of transcripts for our analysis, we identified considerable differences as well as surprisingly analogous characteristics between the examined tissues of humans and Drosophila.

## 2. Results

### 2.1. Expression Patterns and Tissue Specificity of Human and Drosophila Tissues

#### 2.1.1. A Considerable Portion of Transcripts Are Showing Tissue Enrichment in Both Human and Drosophila

**Figure 1:**
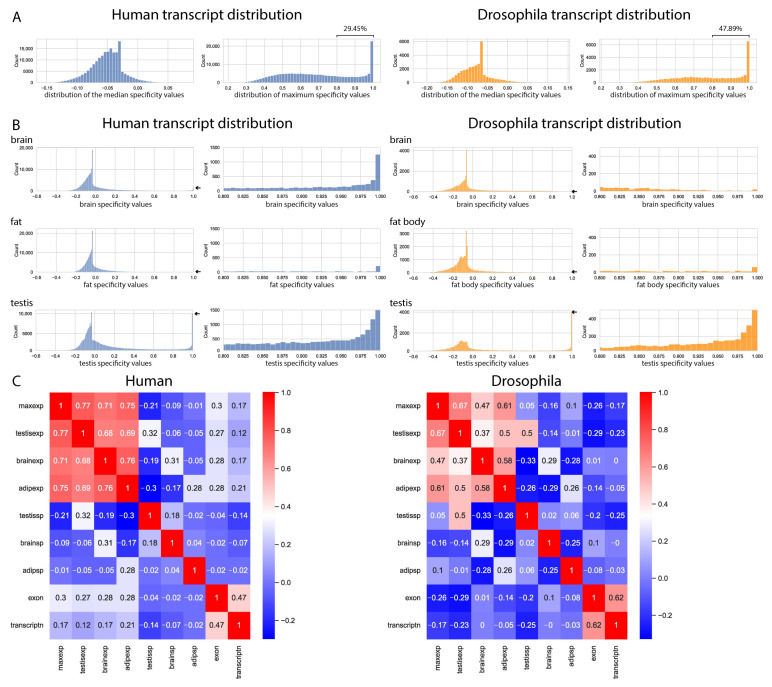
Transcript enrichment profiles (**A**) Histograms represent the distribution of median specificity values and maximum specificity values of human (blue) and Drosophila (orange). (**B**) Histograms represent the distribution of transcripts based on their tissue specificity in human (blue) and Drosophila (orange). Brain, adipose tissue, and testis are presented. Next to the entire tissue profiles, the distribution of the highly enriched (specificity > 0.8) transcripts is displayed. Black arrows indicate the peak of tissue-specific transcripts. (**C**) Heatmaps represent Spearman correlation coefficients based on maximum expression values (maxexp), tissue expression values (testisexp, brainexp, adipexp), specificity values (testissp, brains’, adipsp), exon numbers (exon), and the transcript number of the corresponding gene (transcriptn).

In Drosophila, numerous genes that exhibit testis-specificity or enrichment have been reported [62]. Thousands of genes demonstrate substantial enrichment in the Drosophila testis, contrasting with human, where the overall number of testis-enriched genes is notable, but the subset of genes exhibiting high levels of testis enrichment is relatively modest (compared to Drosophila), estimated at around 240 [9,40,63]. This phenomenon is surprising given the high number of tissue-specific genes in vertebrates [9]. To investigate this phenomenon, transcriptional data from FlyAtlas2 and GTEx databases were collected. The study centered on transcript variant enrichment instead of gene enrichment within the investigated tissues of both organisms. Examining the transcript variants offers several advantages: it allows for the identification of the transcription start site (TSS) for each transcript, and related sequences can be gathered, leading to a more accurate analysis. Additionally, it distinguishes transcript variants of the same gene and allows the investigation of differently expressed transcripts. We calculated and normalized the tissue specificity index of transcripts based on publicly available transcriptomic data [64], where a value of 1 represents tissue-specific transcripts (Supplementary Table S1 and Figure S1). The distribution of median values of tissue specificity and maximum values is similar in both human and fly (Figure 1A, Supplementary Figure S2A). Based on maximum specificity values, the highly enriched (above 0.8 tissue specificity index) transcripts account for 29.45% of the total human transcripts and 47.89% of the total Drosophila transcripts, suggesting Drosophila has proportionately more highly enriched transcripts in different tissues (Figure 1A). This also means that in human tissues, ~70% of the transcripts have considerable expression in multiple organs.

#### 2.1.2. Investigating Tissue Specificity Revealed an Extreme Number of Testis Enriched Transcripts

Based on the tissue enrichment analyses by Mantica and colleagues [9], one could expect a high number of tissue-specific transcripts in the brain and testis and fewer tissue-specific transcripts in adipose tissues. The investigation of these transcripts revealed an extremely high number of testis-specific transcripts compared to any other tissue in both humans (20,420, 9.53% of total transcripts) and Drosophila (6336, 18.89%) (Figure 1, Supplementary Figures S1 and S2B). The following three tissues with the highest amount of tissue-enriched transcripts in humans are the bone marrow (6324, 2.95%), the brain (5457, 2.55%), and the skeletal muscle (3169, 1.48%). Meanwhile, in Drosophila the ovary (1462, 4.36%), the accessory glands (1395, 4.16%), and the midgut (1067, 3.18%) have a relatively higher number of highly enriched transcripts. Based on the calculations, human fat has 1028 (0.48%) highly enriched transcripts, and Drosophila fat body has 415 (1.24%). Further investigation focused on the transcripts of the brain, the adipose tissues, and the testis (Figure 1B). All of these tissues are characterized by specialized lipid metabolism and possess entirely different biological roles. Human fat shows a similar pattern of a low amount of enriched transcripts to those observed in the Drosophila adult fat body (Figure 1B). It is important to emphasize that the testis also shows a similar distribution of an extremely high number of enriched transcripts in both organisms. Remarkably, there is a divergent pattern in the distribution of tissue-specific transcripts within the brain (Figure 1B). In Drosophila, the quantity of brain-specific transcripts is relatively limited when compared to humans, and the overall presence of brain-specific transcripts (specificity values close to 1) is notably low (Figure 1B). This was further investigated as some redundancy is present in the Drosophila dataset. The head and eye data were omitted from the initial dataset to test if the amount of specific transcripts increases (Supplementary Figure S1). Excluding head data and head and eye data considerably increased the number of enriched transcripts in the brain; however, the number of brain-specific transcripts elevated modestly. After the testis and ovary, the brain showed the most distant pattern to the mean median values, suggesting a more specialized gene expression (Supplementary Figure S2B). These results suggest that Drosophila brain has more mildly or highly enriched transcripts but does not contain a lot of specific ones. The presence of neurons in the periphery could explain this pattern and could be further addressed on a cell-level comparison. As a consequence, in Drosophila the specific transcripts of the nervous tissue could be underrepresented in this study.

#### 2.1.3. Expression Patterns Are More Conserved in Human

The potential correlations among expression values, tissue specificity values, and the number of exons and introns across the three tissues were tested (Figure 1C). In human, gene expression correlates between tissues (brain exp.–testis exp.: 0.68, brain exp.–adipose exp.: 0.76, testis exp.–adipose exp.: 0.69), while tissue specificity shows low correlation with the corresponding expression profile (brain exp.–brain sp.: 0.31, adipose exp.–adipose sp. 0.28, testis exp.–testis sp. 0.32). Additionally, testis specificity in human shows low negative correlation to expression values in other tissues (brain exp.–testis sp.: −0.19, adipose exp.–testis sp.: −0.3). In Drosophila, the correlation of gene expression is lower between the tissues (brain exp.–testis exp.: 0.37, brain exp.–adipose exp. 0.58, testis exp.–adipose exp. 0.5), while specificity indices show medium or low correlation to the corresponding expression values (brain exp.–brain sp.: 0.29, adipose exp.–adipose sp.: 0.26, testis exp.–testis sp.: 0.5) and certain specificity values show low negative correlation to other expression values (brain exp.–adipose sp.: −0.29, adipose exp.–brain sp.: −0.28, testis sp.–brain exp.: −0.33, testis sp.–adipose exp.: −0.26). These data suggest that Drosophila gene expression is more diverse between tissues. In both human and Drosophila, there is a correlation between exon number and transcript number; however, this correlation is more substantial in Drosophila. There is minimal negative correlation between transcript number and testis specificity in both humans and Drosophila. In Drosophila testis expression and testis-specificity negatively correlate with exon number. These data suggest that genes that have testis-enriched transcripts have fewer transcript variants, and in the case of Drosophila, they are composed of fewer exons.

### 2.2. Transcript and Gene Characteristics Change Within Specificity Groups

**Figure 2:**
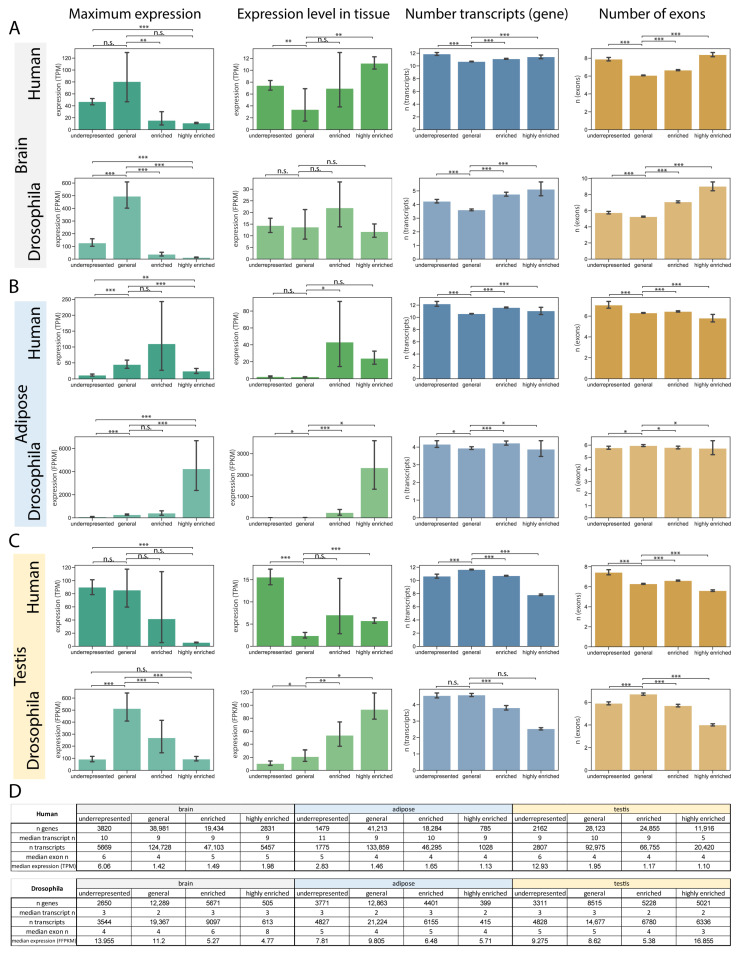
Basic properties of transcript enrichment groups (**A**–**C**) Bar plots represent the mean number of exons, expression levels, and number of transcripts based on testis specificity groups (underrepresented, general, enriched, highly enriched) in the brain (**A**), adipose tissue (**B**) and testis (**C**). Error bars represent 95% confidence intervals. ‘n.s.’ indicates non-significant differences, while ‘*’ denotes *p* < 0.05, ‘**’ indicates *p* < 0.01, and ‘***’ represents *p* < 0.001. (**D**) Additional information on testis specificity groups, including: the number of genes, the median number of genes’ transcript variants, the number of transcripts, the median number of transcripts’ exons, and the median expression of the transcripts.

For additional analysis, the transcripts were organized based on their tissue specificity data, leading to the establishment of four categories: underrepresented transcripts (below −0.20), generally expressed transcripts (between −0.20 and 0.00), enriched transcripts (from 0 to 0.80), and highly enriched transcripts (exceeding 0.80). The distribution of genes and transcripts based on these categories is represented in Figure 2.

It is important to notice specific limitations of the analyses: transcripts that are specific to one tissue may fall into the category of generally expressed transcripts in other tissues. Transcripts that are specific to certain tissues show expression exclusively in one tissue, while remaining unexpressed in others. This suggests a uniform expression pattern, marked by the absence of expression elsewhere, thereby contributing to a broader expression pattern. Underrepresented expression indicates genes that show greater expression in other tissues while exhibiting reduced expression in the tissue of interest. Moreover, the established groups are redundant at the gene level, as genes with different transcripts can be categorized into multiple groups based on the expression of their transcripts. In addition, there might be critical differences attributable to the diverse structure of the genome. The Drosophila genome is more compact and less complex, has fewer repetitive elements, and genes have shorter introns.

#### 2.2.1. Brain Characteristics Revealed More Complex Gene Structures Among Genes with Highly Enriched Transcripts

In the context of the brain, the tissue specificity histogram reveals considerable variation between human and Drosophila (Figure 1). In Drosophila, the number of transcripts exclusively expressed in brain is surprisingly low, this might be an artefact, as head sequence data was also utilized, which artificially lowered the presence of the specific transcripts. Omitting data from the adult head increased the overall number of enriched transcripts but showed similar patterns as before (Supplementary Figure S1). Therefore, we hypothesize that the peripheral nervous system might influence the transcriptomic results of different tissues.

We examined the expression levels (highest expression of the transcript and expression within the tissue), as well as the numbers of transcripts (genes) and exons, utilizing the tissue specificity groups that were defined according to brain-specificity indices. The expression pattern based on the maximum expression of a transcript is similar between human and Drosophila; yet the highly enriched transcripts exhibit increased expression in the human brain (Figure 2A,D). The transcript number is mildly higher in the highly enriched category compared to the general category. The highly enriched transcripts in the brain have an overall higher exon number (Figure 2A,D). Based on these data, it seems the brain utilizes a more complex gene pool, with higher potential transcript variability.

Enriched gene ontology (GO) terms were determined for each specificity category in both organisms. In the highly enriched category, the top 10 terms are mainly related to neuronal functions, confirming that the highly enriched transcripts likely serve functional roles in the brain (Supplementary Figure S3 and Table S2). Interestingly, the GO terms in all other categories in both organisms are related to spermatogenesis. These terms mostly highlight cytoskeletal elements (dyneins) and cilia. The binary nature of cilia-related terms in both enriched and underrepresented categories suggests that some cytoskeletal components associated with motile cilia are present in neurons, while others are absent. These differences could be explained by the crucial functions and high variety of cilia in the nervous system [65]. Cilium varieties might require more specialized protein components, which could be provided with specific transcript variants.

#### 2.2.2. Transcripts Enriched in Adipose Tissue Have Higher Expression in the Tissue

In adipose tissue, the mean expression levels of enriched and highly enriched transcripts are typically elevated, indicating a higher expression level in the tissue; however, the median expression levels are lower (Figure 2B,D). The number of transcripts and exons varies slightly across different categories of adipose tissue. Notably, in humans, the exon count within the highly enriched category is marginally reduced (Figure 2B,D).

GO term analyses on genes that have transcripts enriched and highly enriched in adipose tissue return expressions related to metabolism and lipid metabolism. However, in Drosophila, these terms are not as clearly represented as in humans. Moreover, at lower scores, a variety of other GO terms are enriched in human (Supplementary Table S2 and Figure S3).

#### 2.2.3. Genes with Testis-Enriched Transcripts Have Simpler Genomic Organisation in Both Human and Drosophila

The highest number of specific transcripts can be observed in the testis of both human and Drosophila (Supplementary Table S1, Figure 1). The maximum expression values in human show that highly enriched transcripts in testis exhibit a relatively low expression. The examination of gene expression within the testis indicates that the mean expression of widely expressed transcripts is the least among the categories. Conversely, in humans, the underrepresented category demonstrates the highest expression levels and the largest number of exons. One notable difference between humans and Drosophila is that the genes enriched in the testis of Drosophila generally demonstrate higher expression levels than those observed in human testis (Figure 2C,D). In both human and Drosophila, the highly enriched transcripts have the fewest exons, and genes have the fewest transcripts compared to other categories. These data suggest that highly enriched genes in the testis usually have a simpler gene structure.

In both human and Drosophila, the highly enriched categories’ GO enrichment analyses show GO terms related to meiosis and spermatogenesis (Supplementary Table S2 and Figure S3). Additionally, in human more GO terms are related to cytoskeletal structures and motility. On the other hand, in Drosophila, cytoskeleton-related GO terms are present, and metabolic and protein homeostasis-related terms also appear. In the enriched groups, the additional terms could be easily associated with spermatogenesis and related processes. In human data the metabolism-related terms similarly show an enrichment. An interesting finding in these groups is that in both organisms, the term “nervous system development” occurred (Supplementary Figure S3). Furthermore, it is important to highlight that the analysis of Gene Ontology (GO) terms reveals a significant presence of unclassified genes among the highly enriched transcripts in the human testis. This observation suggests that the members of this category remain poorly characterized in humans. This also raises the possibility of the existence of crucial, yet unknown testis-specific genes.

### 2.3. Investigating Orthologous Genes with Enriched Transcripts Between Human and Drosophila

#### 2.3.1. Orthologues in Human and Drosophila Have a Similar Expression Pattern Only in Adipose Tissue

After evaluating the general characteristics of different tissues, our next aim was to assess the potential correlations between orthologue genes and their associated transcripts (Supplementary Figure S4 and Table S3). We integrated the Drosophila and human data by referencing the orthologue tables from FlyBase.

Analysis of the unfiltered orthologue data indicated a correlation in human gene expression data across human and Drosophila tissues (Supplementary Figure S4). Upon filtering the data using DIOPT scores exceeding 12, representing a relatively strict cutoff, a correlation in Drosophila gene expression values was increased, leading to the emergence of comparable patterns as noted earlier (Supplementary Figure S4). Next, we selected the highly enriched transcripts from the different tissues of both organisms and examined their correlation. The highly enriched transcripts in the brain show no considerable correlation between Drosophila and human, except for transcript and exon number, which show a low and moderate correlation, respectively (Supplementary Figure S4). Regarding the correlation between the highly enriched categories of the testis, a similar pattern emerged, with no outstanding correlation, although exon number shows a low correlation (Supplementary Figure S4). In contrast, in adipose tissue, the expression of the highly enriched human genes shows an intermediate correlation with Drosophila specificity values (Supplementary Figure S4). These data suggest that, in contrast to the brain and testis, adipose tissues have a more conserved gene expression pattern (Supplementary Figure S4). The lack of a similar pattern to adipose tissues in the testis might be attributed to the higher number of transcripts in these categories, which increases the background and produces noise. However, applying an expression filter with the value of 5, which filters low-expressing transcripts out, does not alter the main pattern considerably. This suggests that in more complex organs, the expression profiles are adapted according to their complexity, which could result in the different gene expression patterns observed in the brain and testis. However, enhancing the resolution by examining single-cell transcriptome data may reveal greater similarities.

#### 2.3.2. Orthologous Genes May Have Highly Enriched Transcripts in Multiple Tissues

**Figure 3:**
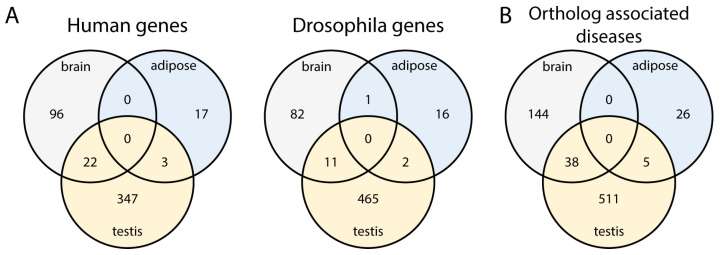
Distribution of genes with highly enriched transcripts (**A**) Venn diagrams represent the distribution of orthologous genes that have highly enriched transcripts in different organs. (**B**) The Venn diagram represents the number of ortholog associated diseases (OMIM—Flybase disease annotations) across different tissues.

Based on the transcript enrichment of orthologous genes, we were interested in whether there are genes that have highly enriched transcripts in different organs (Figure 3A). In humans, 22 (18.64%) genes that have highly enriched transcripts in the brain also have highly enriched transcripts in the testis; in Drosophila, there are 11 (11.7%). Some genes have highly specific transcript variants in tests and adipose tissue, both in humans (3) and Drosophila (2). The brain and adipose tissue have no overlap in humans, whereas the brain and testis have a proportionately similar overlap with adipose tissue. These data suggest that among the highly enriched orthologous genes, the brain and adipose tissue are more diverged.

#### 2.3.3. Orthologous Genes with Highly Enriched Transcripts Could Serve as Disease Models

Gene orthologues provide a good target for studying their potential implications in diseases, and therefore, we investigated the human-associated diseases linked to the highly enriched orthologues. In our dataset, 30.8% (3859/12,528) of the Drosophila genes have human orthologues with disease associations based on OMIM. Meanwhile, the analysis of highly enriched orthologous transcripts from humans and Drosophila reveals that 37.1% (118 out of 318 genes) are associated with a disease in the brain data, 50% (20 out of 40 genes) with diseases linked to adipose tissue, and 28.2% (372 out of 1318 genes) associated with conditions in the testis data. The diseases in brain cover a wide variety of conditions, mainly related to brain development and function (Supplementary Table S4). The adipose tissue dataset shows a variety of diseases, including cancer and developmental disorders. In the testis dataset, a wide spectrum of diseases is also present, with spermatogenic failure occurring in 24 cases. Many disease-associated genes encode cytoskeletal elements (dynein, actin), but enzymes (kinases, phosphatases), chaperones, and RNA helicases are also present (Supplementary Table S3). We investigated the highly enriched orthologous genes that are associated with diseases, and the overlap of these diseases between tissues (Figure 3B, Supplementary Table S4). Thirty-eight diseases are associated with both the brain and testis, and five in the testis and adipose tissue. There is no overlap between the brain and adipose tissue. These data suggest that a pleiotropic effect could be expected among genes that have highly enriched transcripts in multiple tissues, and that not only generally expressed transcripts can contribute to pleiotropy.

### 2.4. Network Analysis Revealed Conserved Central Elements Are Present in All Tissues

**Figure 4:**
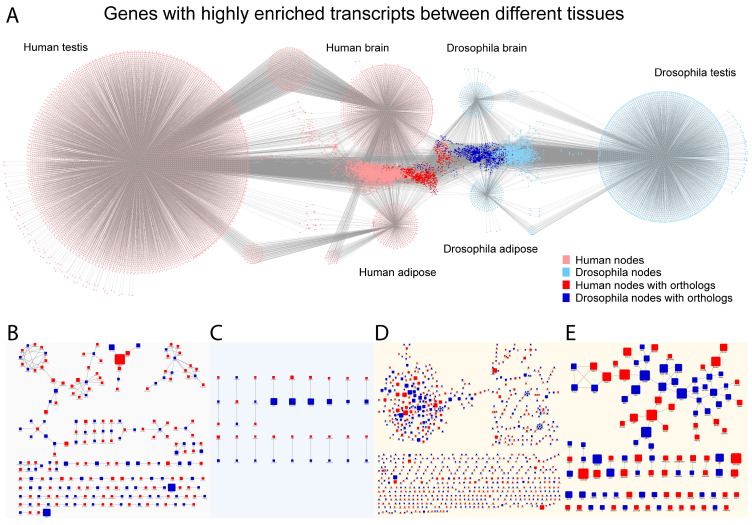
Network representation of genes with highly enriched transcripts Network models representing genes with highly enriched transcripts in different tissues and their known physical interactions. The tissue-enriched genes have a limited number of known interactions; therefore, the connections are likely underrepresented. (**A**) Human tissue-related genes are represented with red shading, and Drosophila tissue-related genes with blue shading. Gene nodes with orthologues are colored in red (human) and dark blue (Drosophila), genes with no orthologues are pink (human) and light blue (Drosophila. (**B**) Orthologous genes network in the brain (edges represent physical interactions and connect orthologues) (**C**) Orthologous genes in adipose tissue (edges connect orthologues). (**D**) Orthologous genes in the testis with known physical interaction (edges represent physical interactions and connect orthologs). (**E**) Subset of nodes from the testis with more than 20 known physical interactions.

Network analysis is a powerful tool to identify important components of complex systems. We established network models using genes that have highly enriched transcripts in the brain, adipose tissue, or testis. Additionally, we mapped the known physical interactions between gene products (from FlyBase and Human Protein Atlas). Moreover, we linked the human and Drosophila network based on orthology (Figure 4A, Supplementary Figure S5 and Supplementary File S1). In both human and Drosophila, the testis has the most genes associated with it and the most gene nodes shared with other tissues. The mapped physical interactions form a central network that shows the connection to the investigated tissues; moreover, orthologs are enriched in these highly connected regions.

Tissue-by-tissue comparison showed that in adipose tissue, there are orthologue pairs with fewer known physical interactions, while in the brain and testis, more connected subnetworks are also present.

Investigating only the orthologous genes, a more extended network was observed in the brain and testis, but not in adipose tissue (Figure 4B–E). Additional filtering by degree (of physical interactions > 20) revealed some interesting nodes in all three tissues. In the brain, a single orthologue pair remained: the human YWHAG and the Drosophila orthologue 14-3-3zeta. Similarly, to the brain, in adipose tissue only a single ortholog pair remained after filtering: the human PPP2R1B and the Drosophila Pp2A-29B. In contrast, the testis network showed numerous orthologous gene pairs after filtering (Figure 4E). These pairs mostly include transcription factors (TP53-p53), ubiquitin–proteasome system components (cullins, proteasome subunits), kinases (Aurora kinase), phosphatases (Pp2A-29B), and RNA-binding proteins (FMR1, EIF4E) (Figure 4E, Supplementary Table S5). The gene pairs identified are strong candidates for further investigation based on their conservation and central position. However, it is important to note that network building relies heavily on physical interaction data that do not take into account tissue-specific connections and interactions. Additionally, tissue-specific genes and isoforms are less studied. Therefore, the presence of tissue-specific connections and subnetworks is likely underrepresented.

### 2.5. Sequence Analysis of Enriched Transcripts Showed Characteristic Patterns

#### 2.5.1. Transcriptional Start Site Regions Show Characteristic ATGC Content

One of the benefits of conducting research at the transcript level is that annotated transcript sequences can be acquired and directly aligned with other data.

**Figure 5:**
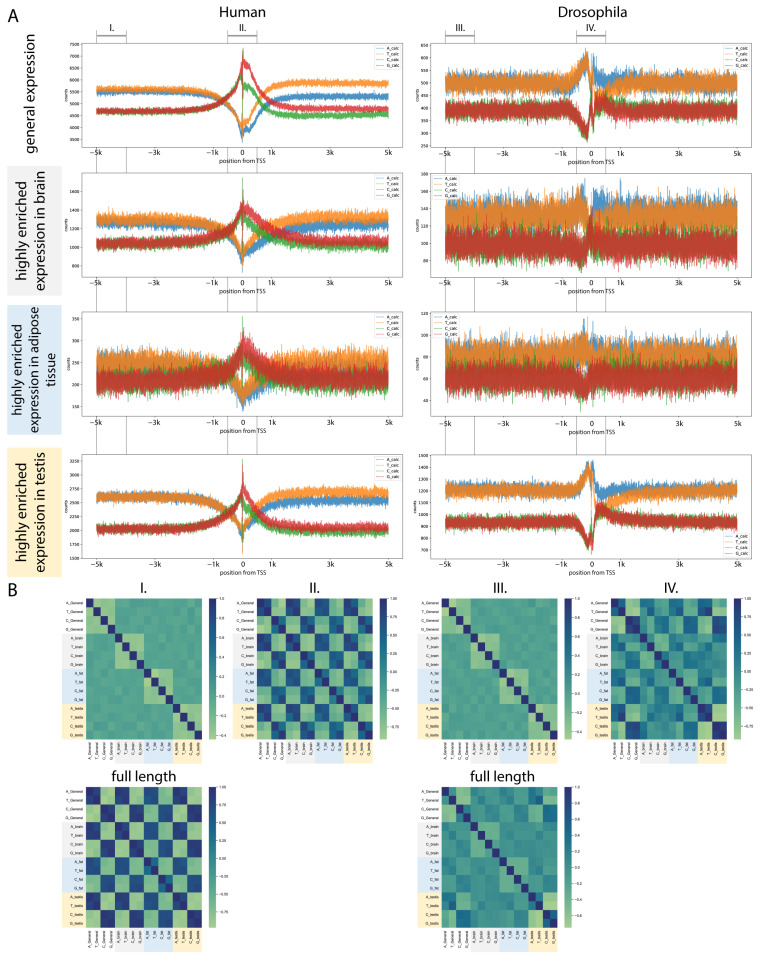
Nucleotide base distribution around TSSs of highly enriched transcripts (**A**) Graphs represent the ATCG base distribution in a 10 kb region around the transcriptional start sites of generally expressed genes (filtered: have no highly enriched transcript), and transcripts highly enriched in the brain, adipose tissue, and testis. (**B**) Heatmaps represent the correlation of bases (ATCG) between the base counts of differently enriched transcripts across upstream (I, III), TSS (II, IV), and full-length sequences.

We collected the highly enriched transcripts from the brain, adipose tissue, and testis, and extracted unique genomic sequences upstream and downstream of the first nucleotide of the annotated transcripts. A control group was also established using transcripts that do not show high enrichment in any tissue in either human or Drosophila. We examined the distribution of nucleotide bases—adenine (A), thymine (T), cytosine (C), and guanine (G)—and noted that their distribution exhibits a distinct profile in the vicinity of TSSs [66] (Figure 5, Supplementary Figure S6).

#### 2.5.2. Human TSS Shows High Similarity Between Tissues

In humans, the distribution of AT and GC content exhibits similarities in the regions upstream of the TSS. However, closer to the TSS, there is a notable increase in GC content, accompanied by a decrease in AT content. Following the transcription start site (TSS), there is an increase in the AT content while the GC content declines. Notably, downstream of the TSS, the nucleotides thymine (T) and guanine (G) are found to be considerably more prevalent than adenine (A) and cytosine (C). This phenomenon is attributed to the transcription-associated repair mechanisms [67]. Next, the transcripts exhibiting high tissue enrichment were analyzed, and a comparable trend was noted across all three examined tissues and the control group (Figure 5, Supplementary Figure S6).

Examining the TSS region at higher resolution, other interesting patterns emerge. In humans, at the TSS, ~25 bp upstream, an AT peak is observable, likely corresponding to the TATA box. In the investigated tissue data, ~25 bp downstream of the TSS, a peak of A and G is present, while the amount of T and C is lower (Supplementary Figure S6).

#### 2.5.3. The TSS of Drosophila Tissue-Enriched Transcripts Show Different ATGC Profiles Between Tissues

In Drosophila, the base profile differs considerably from humans, but upstream of the TSS, the pattern is similar. The region surrounding the TSS shows a rise in AT content in contrast to the human genome, with the GC content displaying two notable peaks. Downstream of the TSS, the A content is considerably greater than T for approximately 500 base pairs. However, the intergenic pattern recovers in Drosophila after this, while in human, the upstream intergenic pattern does not recover within 5 kbp. The investigated tissues exhibit much lower similarity in base distribution. Testis-enriched transcripts show the most similar pattern to the generally expressed genes. (Figure 5, Supplementary Figure S6)

In brain-enriched sequences, the ~100 bp upstream region, the AT and GC content are almost equal, but the T peak and the overall increase in the amount of A after the TSS are present. Additionally, the amount of G also seems to be higher downstream to the TSS. In fat bodies, the T peak is observable near the TSS, and there is a higher amount of A downstream of TSS. The testis dataset shows that the AT content is not dropping considerably at the TSS, as only C numbers elevate mildly. The higher T peak is absent downstream of the TSS; instead, there is a peak upstream of the TSS. The TSS region of highly enriched genes in Drosophila testis shows the highest AT content. (Supplementary Figure S6)

#### 2.5.4. ATGC Profile of Non-Coding RNA TSS Varies in Drosophila Testis

We also investigated the ATCG profile around the TSSs of non-coding RNA genes. In brain and adipose tissues, the number of sequences was too low to plot; therefore, we focused on the testis. The TSS region of non-protein coding genes enriched in the testis and the TSS regions of all other non-coding RNAs were plotted (Supplementary Figure S7). The non-coding RNAs’ TSS region ATCG profile is less diverse. In humans, non-enriched testis non-coding transcripts have a profile where GC peak, and there is an elevated amount of T downstream of the TSS, similar to the transcript profile presented earlier. In contrast to this, the testis-enriched transcripts also show the GC elevation, but downstream of the TSS, the T enrichment is not characteristic, and a low C content is observable. This raises the possibility that the non-coding RNA pool in the testis is evolutionary younger, as the transcription-associated repair did not considerably shift the T content. In Drosophila, the ATCG profile of non-coding RNAs that are not enriched in testis around the TSS exhibits fewer characteristic changes. The TSS has peaks, but the upstream and downstream regions are quite homogeneous. In contrast, the testis-specific non-coding RNAs show an elevated amount of AT both upstream and downstream of the TSS, a considerable G peak at the TSS, and an elevated GC content downstream of the TSS. This profile is different from the one that was observed previously with highly enriched transcripts.

### 2.6. Differentially Enriched Motifs Are Present in the TSS Region of Enriched Transcripts

Next to ATGC profiling the sequences associated with enriched transcripts, the sequences could be investigated further based on conserved elements.

We investigated the highly enriched transcripts’ DNA sequences upstream and downstream of the TSS using the MEME Suite to find differentially enriched motifs. Three sequence segments were analyzed: the upstream 300 bp; and the core promoter region, tested by splitting it into 100 bp upstream and 100 bp downstream sequences relative to the TSS. Sequences of the highly enriched transcripts were investigated in the brain, adipose tissue, and testis (Supplementary Figure S8).

We found differentially enriched motifs in almost all categories. Most motifs show high sequence variety and can be associated with numerous transcriptional factors using Tomtom for comparison. In humans, brain-enriched transcripts show the most conserved motifs, which can be associated with Patz1, SP1, and WT1 transcription factors. Human adipose tissue transcript sequences present GA-enriched motifs that are present in all of the investigated sequence sets. We found the highest number of differentially enriched motifs in the human testis; these motifs can be associated with CPEB1, NFAC2, and SOX2 recognition sites. The most interesting motif is almost identical to the RFX family transcription factor recognition sites.

In Drosophila, a similar search for motifs was conducted. Motifs found in genes that are highly enriched in the brain included Eip74EF, Mad, HLH4C, wor, Adf1, and CG12605 transcription factors. HLH4C, wor, and CG12605 are known for their role in the nervous system. The adipose dataset in Drosophila shows no differentially enriched DNA motifs. In the testis, the upstream 100 bp region shows the presence of more differentially enriched motifs than the 300 bp region. We found motifs that can be associated with vis, achi, hth, exd, fru, retn, vvl, Antp, Hr51, and br. Based on FlyBase, the vis and Achi are known to be required for spermatogenesis. The hth and exd form a complex and they are Hox cofactors. Their role in spermatogenesis is not clarified; nevertheless, both of their recognition motifs are present in the upstream 100 bp of highly enriched transcripts. Fruitless function is known in sexual differentiation. The transcription factors coded by *retn*, *vvl*, *Antp*, *Hr51*, and *br* are known for regulating developmental processes. Investigating the downstream 100 bp sequences of testis-enriched transcripts, we identified 2 enriched motifs. Interestingly, one of these has no hits using Tomtom; the other one can be associated with Pho and Phol. Pho is believed to be primarily a transcriptional suppressor; however, the GC-rich first segment of the motif is missing.

The investigation of human tissues revealed a higher number of differentially enriched patterns in the upstream 300 bp sequences, similar to the Drosophila brain dataset. Meanwhile, the Drosophila testis dataset exhibits the most motifs in the upstream 100 bp region. Based on this, we hypothesize that the Drosophila gene expression in the testis relies more heavily on the core promoter region.

## 3. Discussion

There are numerous genes that were established by retro-position in Drosophila that are predominantly expressed in the testis, contributing to the high number of testis-specific genes. These retrogenes have few or no introns and encode only a few transcript variants. Retrogenes in Drosophila and human often gain testis-enriched expression, which increases the number of testis-specific genes [68,69,70,71,72]. De novo gene expression also tend to occur in testis [73,74]. The properties of retrogenes and de novo genes are reflected in our study, where we present a lower number of exons and transcripts in testis-enriched genes. In humans, functional retrogenes are less common, yet a similar gain of testis-specificity occurs among human genes. Despite this, the number of testis-specific genes is relatively lower in humans compared to Drosophila. However, as we present here, the number of testis-enriched transcripts is extremely high in both organisms. The lower number of testis-specific genes can be explained by the higher number of transcripts in humans, as the different transcripts may express in different tissues, thereby gaining expression in multiple tissues. The high number of transcripts is considered to be connected with the higher complexity observable in mammals [75,76]. Our investigation of the transcriptional start site showed that in human, the DNA base profile of enriched transcripts is more conserved than that observed in Drosophila. This could also explain why there are fewer testis-specific genes and suggests the more important role of regulatory elements and epigenetics in humans. This feature also explains the transcript expression patterns: in humans, a more general expression is present, while Drosophila expression profiles show more diversity between tissues, reflected by a more diverse base distribution pattern around the TSS. The differences observed between tissues in Drosophila are particularly evident when comparing the brain and testis base profile of the TSS. Transcripts that have highly enriched expression in the brain show an increased GC content upstream of the TSS, whereas in testis, AT content is much higher. This suggests that GC nucleotides or GC-rich motifs may play a role in brain gene expression regulation but have no importance in the testis. Interestingly, a lack of CpG islands was previously demonstrated in 50% of human testis-specific genes [40].

The AT-rich testis core promoter in Drosophila might influence gene expression based on its physical properties. Poly-AT sites are intrinsically rigid structures that reduce nucleosome formation likelihood. Moreover, in yeast, AT-rich promoter sequence was observed in constitutively active genes [77,78,79]. This suggests that in the highly relaxed DNA found in Drosophila spermatocytes, the transcription could be initiated through more accessible promoter regions.

The correlation of gene expression, the similarity of DNA base profiles between human tissues, and the differentially enriched DNA motifs found in the promoter region strongly suggest that human testis-specific expression relies mostly on enhancers and epigenetic regulation. The similar genomic organization observed around the transcriptional start sites could explain the more similar expression patterns. In Drosophila, gene expression seems to be more tissue-specific because of the limited correlation between expression values and the physical properties of transcriptional start sites, based on the DNA base profiles. Our results indicate that the upstream 100 nucleotide sequences of the TSS play an important role in transcription initialization in testis.

Despite both Drosophila and human showing an extreme number of testis-specific transcripts, the Drosophila testis-specific genes generally exhibit higher expression levels, which is less typical of human testis-specific transcripts. Moreover, in humans, we can observe the relatively higher expression of underrepresented transcripts, suggesting a potential mechanism of silencing activity in the testis. Overall, a general permissive gene expression is observed in both human and Drosophila but human gene expression appears to be under stricter negative regulation based on expression levels.

Interestingly, highly enriched non-coding RNAs have different DNA base profiles around their TSSs as well. In Drosophila testis, compared to all non-coding RNAs and to testis-enriched transcripts, the testis-enriched non-coding RNA sequences show characteristic differences. The base pattern in humans near the TSS is largely consistent; nonetheless, a notable difference is observed in the testis, characterized by the lack of enrichment of G and T. This suggests that either transcription-associated repair does not work in the testis, or testis-enriched non-coding RNAs are evolutionarily young.

The number of transcripts and exons is higher in brain-enriched genes compared to testis-enriched genes. This finding supports the theory that the testis serves as an evolutionary testing ground for genes, as evolutionary younger genes might have fewer transcripts. The lower exon number could facilitate mature mRNA synthesis, as fewer introns need to be spliced, whereas a higher exon number could provide a larger variety of proteins, which could be beneficial for complex tissues. Despite the differences in gene structure, similar biological processes occur in the brain and testis. The storage, transport, and availability of mature RNA are essential in both tissues [80]. Additionally, the GO annotation revealed the similarity of cytoskeletal elements between brain and testis-enriched transcripts. This might be associated with the crucial role of cilia in neuronal development and signaling [81]. Also variety of cilia types are present in neuronal cells, which might require different molecular structures to function properly [65]. However, further investigation is required to clarify this.

As expected, adipose tissue has the fewest genes with enriched transcripts; these genes displayed a simpler gene structure than what is seen in the brain, yet a more complex structure than that of the testis in both organisms. Since adipose tissue possesses intermediary traits, it can function as a baseline for additional comparisons; unfortunately, the low number of enriched transcripts compromised the reliability of these comparisons. However, enriched orthologs showed an exciting correlation between Drosophila and human. This is noteworthy since Drosophila has already been employed in the study of obesity and its associated disorders [82,83,84].

Gene Ontology analyses showed the potential role of the enriched genes in the given tissues, but also highlighted the similarities between these tissues, namely the structural components that are used in both the brain and the testis. Unfortunately, in the human dataset, the “unclassified” category was enriched in many GO term analyses, suggesting many of the genes with highly enriched transcripts are not yet well characterized.

While our analysis revealed a lack of substantial correlations in the data of orthologous genes, we determined a number of orthologs for every tissue that has highly enriched transcripts. Furthermore, the network analyses revealed that these orthologues tend to exhibit a higher number of physical interactions, suggesting central roles in biological processes. Additionally, we collected associated diseases; investigating genes from these lists could be a good starting point for future research. Network analysis proves to be a powerful tool for identifying conserved elements in developmental programs; for instance, conserved elements of male germ cell development have been identified between humans and *Drosophila* [85]. Our findings also present potential candidates for further experimental investigation.

While there are differences in gene expression between mammals and insects, these findings show the possibility of distinct molecular solutions to achieve similar functional strategies. This underscores the critical adaptations observed in response to shared evolutionary pressures across different tissues and species.

### Limitations of the Study

One of the limitations of the study is the overlapping categories in the investigated tissue types. Some tissues share considerable similarities, like the different regions of the gut, which decrease the number of tissue-specific genes as they might occur in multiple categories. Moreover, some tissues are composed mostly of uniform cells, such as fat, while others consist of considerably different major cell populations, as in the brain. Therefore, comparisons of cellular gene expressions would be more accurate.

Another limitation is the limited knowledge of tissue-enriched genes. As their expression pattern is limited, their interaction network is not well characterized, and many interaction screens use cultured cells that are sensitive only to the gene expression of that cell type. Additionally, GO annotations may also be limited as tissue-specific genes are less studied therefore GO predictions might be inaccurate.

## 4. Materials and Methods

### 4.1. Data Source and Preparation

Drosophila tissue expression data were collected from FlyAtlas2 (https://flyatlas.gla.ac.uk/FlyAtlas2/ (accessed on 13 September 2023)) [86]. Human data was collected from GTEx database (https://www.gtexportal.org/ (accessed on 14 September 2023)). When multiple tissue datasets were available from the same database, the mean expression values of the tissues were utilized for further analyses (FPKM and TPM).

Gene expression levels between organisms were not investigated directly, the formula of Li et al. 2014 was used to determine tissue specificity [62,64].

$$x_{i}=\frac{e_{i}-\bar{e}}{\sqrt{\frac{1}{n-1}\sum_{i=1}^{n}{\left(e_{i}-\bar{e}\right)}^{2}}},$$

where *x* is tissue specificity, *e* is the sample tissue expression value, $\bar{e}$ is the mean gene expression value and *n* is the number of investigated tissues sample from $i=1,\ldots ,n^{th}$. Total number of human tissues was *n* = 33, and Drosophila tissues *n* = 17.

After calculation, the indexes were normalized between −1 and 1, where 1 represents the highest specificity using sci-kit learn’s MaxAbsScaler.

The brain, adipose tissue, and testis were selected for comprehensive analysis from both organisms.

Orthologues and disease data were collected from FlyBase (FlyBase.org), based on DIOPT and the Online Mendelian Inheritance in Man (OMIM, https://omim.org/ (accessed on 25 October 2023)) [51,87,88,89]. Physical interactions were collected from FlyBase and Human Protein Atlas [51,90]. DIOPT scores the orthologues based on their occurrence in 14 individual databases. We investigated orthologues that were present in at least 12 databases.

Computer codes were implemented in Python 3.8. Pandas (v 1.2.4), NumPy (v. 1.22.4), Scikitlearn (v 1.3.2), and SciPy (v 1.6.2) libraries were used for processing data, and Seaborn (v 0.11.1) and Matplotlib (v 3.3.4.) were used for generating graphs. Relevant code is available at: https://doi.org/10.5281/zenodo.14388393 (accessed on 12 December 2024.).

### 4.2. Gene Ontology Analysis

Gene ontology (GO) analysis was conducted to identify the biological processes, molecular functions, and cellular components associated with the gene set under investigation. For this purpose, PANTHER 19.0 (Protein ANalysis THrough Evolutionary Relationships) was utilized via the web-based GO enrichment tool (https://www.pantherdb.org/ (accessed on 29 November 2023)) [91,92,93]. The input genes were mapped to the corresponding GO terms using PANTHER’s reference gene set.

### 4.3. Network Analysis

In this study, we conducted a comprehensive network analysis to identify tissue enriched gene products with physical interactions in the brain, adipose tissue, and testis of human and Drosophila melanogaster. Our approach integrated gene enrichment data, protein–protein interaction (PPI) data, and orthologue data to explore key elements involved in tissue-specific functions.

Cytoscape (Version 3.10.1) [94,95] was used to construct interaction networks through a combination of highly enriched gene set data (filtered tissue specificity), physical interaction data and orthologue data for both organisms. Network centrality measures-including degree, betweenness, and closeness- were applied to the physical interaction data to identify hub genes that are highly connected and potentially critical for tissue function. To highlight the nodes with the most connections a degree filter of 20 was used.

The network structures and identified genes across brain, adipose, and testis tissues in both species, could highlight conserved and tissue-specific patterns. This approach allowed us to predict the potential evolutionary conservation of tissue-specific gene networks between Drosophila and humans.

### 4.4. Transcription Start Sites and Motif Analysis

Transcription start sites were determined based on transcript annotation coordinates in both human and Drosophila. Human coordinates were extracted from gencode annotation file (gencodev46), while Drosophila sequence coordinates were collected from FlyBase precomputed files. Based on these coordinates, genome sequences were extracted from the reference genomes (GRCh38.p14, Dmel_Release_6). Extracted sequences were randomly chosen and validated by using BLAST (v 2.15.0) [96].

MEME Suite 5.5.7 was used for motif discovery (https://meme-suite.org (accessed on 3 September 2024)) [97]. MEME algorithm’s differential enrichment mode with two motif distribution options (zero or one occurrence per sequence, and any number of repetitions) was utilized to find 10 motifs between 6 and 12 nucleotides with a minimum occurrence of 10 sites in each run. Motifs showing statistical significance (default E-value cutoff) were collected. Motifs were searched in transcripts that are highly enriched in the given tissue. The background list was established by selecting genes whose transcripts lacked highly enriched transcript variants, and it was used for every comparison.

Motifs identified were compared to human (HOCOMOCO v11) and Drosophila (combined: OnTheFly_2024, Fly Factor Survey, FLYREG, etc.) databases using MEME Suite’s Tomtom.

### 4.5. Quantification and Statistical Analysis

The significance between specificity groups was assessed using Welch’s two-tailed t-test. Pearson and Spearman correlation coefficients and related *p*-values were calculated using the SciPy (v 1.6.2) library, and the results were visualized as heatmaps using Seaborn (v 0.11.1). Spearman correlation was utilized when transcription data was included. Mean squared error and Euclidean distance were calculated using NumPy (v 1.22.4). The statistical significance of Gene Ontology (GO) term enrichment was evaluated through Fisher’s exact test, with multiple testing corrections applied via the False Discovery Rate (FDR) method to control for Type I errors. GO terms with an adjusted *p*-value of less than 0.05 were deemed significantly enriched and considered for further biological interpretation. The findings were visualized using bubble charts to highlight the most enriched GO categories and were summarized in a datasheet for comprehensive analysis.

## Data Availability

This paper analyzes existing, publicly available data (https://flyatlas.gla.ac.uk/FlyAtlas2/ (accessed on 13 September 2023); https://www.gtexportal.org/ (accessed on 14 September 2023)), processed relevant data is available from Supplementary Materials. All relevant original code and Supplementary Tables have been deposited at Zenodo and is publicly available at https://doi.org/10.5281/zenodo.14388393 (accessed on 12 December 2024) as of the date of publication. Any additional information is available on request.

## Supplementary Materials

The following supporting information can be downloaded at: https://zenodo.org/records/17628846 (accessed on 18 September 2025).

## References

[1] Hill M.S., Vande Zande P., Wittkopp P.J. (2021). Molecular and Evolutionary Processes Generating Variation in Gene Expression. Nat. Rev. Genet..

[2] Coniglio J.G. (1994). Testicular Lipids. Prog. Lipid Res..

[3] Laurinyecz B., Péter M., Vedelek V., Kovács A.L., Juhász G., Maróy P., Vígh L., Balogh G., Sinka R. (2016). Reduced Expression of CDP-DAG Synthase Changes Lipid Composition and Leads to Male Sterility in *Drosophila*. Open Biol..

[4] Jarvis S., Williamson C., Bevan C.L. (2019). Liver X Receptors and Male (In)Fertility. Int. J. Mol. Sci..

[5] Chao C.F., Pesch Y.-Y., Yu H., Wang C., Aristizabal M.J., Huan T., Tanentzapf G., Rideout E.J. (2024). An Important Role for Triglyceride in Regulating Spermatogenesis. eLife.

[6] Tracey T.J., Steyn F.J., Wolvetang E.J., Ngo S.T. (2018). Neuronal Lipid Metabolism: Multiple Pathways Driving Functional Outcomes in Health and Disease. Front. Mol. Neurosci..

[7] Yang D., Wang X., Zhang L., Fang Y., Zheng Q., Liu X., Yu W., Chen S., Ying J., Hua F. (2022). Lipid Metabolism and Storage in Neuroglia: Role in Brain Development and Neurodegenerative Diseases. Cell Biosci..

[8] Cardoso-Moreira M., Halbert J., Valloton D., Velten B., Chen C., Shao Y., Liechti A., Ascenção K., Rummel C., Ovchinnikova S. (2019). Gene Expression across Mammalian Organ Development. Nature.

[9] Mantica F., Iñiguez L.P., Marquez Y., Permanyer J., Torres-Mendez A., Cruz J., Franch-Marro X., Tulenko F., Burguera D., Bertrand S. (2024). Evolution of Tissue-Specific Expression of Ancestral Genes across Vertebrates and Insects. Nat. Ecol. Evol..

[10] Ahn J., Wu H., Lee K. (2019). Integrative Analysis Revealing Human Adipose-Specific Genes and Consolidating Obesity Loci. Sci. Rep..

[11] Yuan S.-X., Li H.-T., Gu Y., Sun X. (2021). Brain-Specific Gene Expression and Quantitative Traits Association Analysis for Mild Cognitive Impairment. Biomedicines.

[12] Shimada M., Omae Y., Kakita A., Gabdulkhaev R., Hitomi Y., Miyagawa T., Honda M., Fujimoto A., Tokunaga K. (2024). Identification of Region-Specific Gene Isoforms in the Human Brain Using Long-Read Transcriptome Sequencing. Sci. Adv..

[13] Negi S.K., Guda C. (2017). Global Gene Expression Profiling of Healthy Human Brain and Its Application in Studying Neurological Disorders. Sci. Rep..

[14] Huang G., Osorio D., Guan J., Ji G., Cai J.J. (2020). Overdispersed Gene Expression in Schizophrenia. npj Schizophr..

[15] Sun W., Xie G., Jiang X., Khaitovich P., Han D., Liu X. (2023). Epigenetic Regulation of Human-Specific Gene Expression in the Prefrontal Cortex. BMC Biol..

[16] Babenko V., Redina O., Smagin D., Kovalenko I., Galyamina A., Kudryavtseva N. (2024). Brain-Region-Specific Genes Form the Major Pathways Featuring Their Basic Functional Role: Their Implication in Animal Chronic Stress Model. Int. J. Mol. Sci..

[17] McKenzie A.T., Wang M., Hauberg M.E., Fullard J.F., Kozlenkov A., Keenan A., Hurd Y.L., Dracheva S., Casaccia P., Roussos P. (2018). Brain Cell Type Specific Gene Expression and Co-Expression Network Architectures. Sci. Rep..

[18] Janssens J., Aibar S., Taskiran I.I., Ismail J.N., Gomez A.E., Aughey G., Spanier K.I., De Rop F.V., González-Blas C.B., Dionne M. (2022). Decoding Gene Regulation in the Fly Brain. Nature.

[19] Davie K., Janssens J., Koldere D., De Waegeneer M., Pech U., Kreft Ł., Aibar S., Makhzami S., Christiaens V., Bravo González-Blas C. (2018). A Single-Cell Transcriptome Atlas of the Aging Drosophila Brain. Cell.

[20] Malacrinò A., Brengdahl M.I., Kimber C.M., Mital A., Shenoi V.N., Mirabello C., Friberg U. (2022). Ageing Desexualizes the Drosophila Brain Transcriptome. Proc. R. Soc. B Biol. Sci..

[21] Kaessmann H. (2010). Origins, Evolution, and Phenotypic Impact of New Genes. Genome Res..

[22] Kondo S., Vedanayagam J., Mohammed J., Eizadshenass S., Kan L., Pang N., Aradhya R., Siepel A., Steinhauer J., Lai E.C. (2017). New Genes Often Acquire Male-Specific Functions but Rarely Become Essential in *Drosophila*. Genes Dev..

[23] Laurinyecz B., Vedelek V., Kovács A.L., Szilasi K., Lipinszki Z., Slezák C., Darula Z., Juhász G., Sinka R. (2019). Sperm-Leucylaminopeptidases Are Required for Male Fertility as Structural Components of Mitochondrial Paracrystalline Material in Drosophila melanogaster Sperm. PLoS Genet..

[24] Vedelek V., Laurinyecz B., Kovács A.L., Juhász G., Sinka R. (2016). Testis-Specific Bb8 Is Essential in the Development of Spermatid Mitochondria. PLoS ONE.

[25] Alzyoud E., Vedelek V., Réthi-Nagy Z., Lipinszki Z., Sinka R. (2021). Microtubule Organizing Centers Contain Testis-Specific γ-TuRC Proteins in Spermatids of Drosophila. Front. Cell Dev. Biol..

[26] Beall E.L., Lewis P.W., Bell M., Rocha M., Jones D.L., Botchan M.R. (2007). Discovery of tMAC: A *Drosophila* Testis-Specific Meiotic Arrest Complex Paralogous to Myb–Muv B. Genes Dev..

[27] Emelyanov A.V., Barcenilla-Merino D., Loppin B., Fyodorov D.V. (2023). APOLLO, a Testis-Specific Drosophila Ortholog of Importin-4, Mediates the Loading of Protamine-like Protein Mst77F into Sperm Chromatin. J. Biol. Chem..

[28] Zhang X., Peng J., Wu M., Sun A., Wu X., Zheng J., Shi W., Gao G. (2023). Broad Phosphorylation Mediated by Testis-Specific Serine/Threonine Kinases Contributes to Spermiogenesis and Male Fertility. Nat. Commun..

[29] Ahn J., Kim D.-H., Park M.-R., Suh Y., Lee H., Hwang S., Mamuad L.L., Lee S.S., Lee K. (2022). A Novel Testis-Enriched Gene, Samd4a, Regulates Spermatogenesis as a Spermatid-Specific Factor. Front. Cell Dev. Biol..

[30] Morohoshi A., Miyata H., Tokuhiro K., Iida-Norita R., Noda T., Fujihara Y., Ikawa M. (2023). Testis-Enriched Ferlin, FER1L5, Is Required for Ca^2+^-Activated Acrosome Reaction and Male Fertility. Sci. Adv..

[31] Ma Q., Cao C., Zhuang C., Luo X., Li X., Wan H., Ye J., Chen F., Cui L., Zhang Y. (2021). AXDND1, a Novel Testis-Enriched Gene, Is Required for Spermiogenesis and Male Fertility. Cell Death Discov..

[32] Kaneda Y., Miyata H., Shimada K., Oyama Y., Iida-Norita R., Ikawa M. (2022). IRGC1, a Testis-Enriched Immunity Related GTPase, Is Important for Fibrous Sheath Integrity and Sperm Motility in Mice. Dev. Biol..

[33] Li P., Messina G., Lehner C.F. (2023). Nuclear Elongation during Spermiogenesis Depends on Physical Linkage of Nuclear Pore Complexes to Bundled Microtubules by Drosophila Mst27D. PLoS Genet..

[34] Suzuki A., Yabuta N., Shimada K., Mashiko D., Tokuhiro K., Oyama Y., Miyata H., Garcia T.X., Matzuk M.M., Ikawa M. (2024). Individual Disruption of 12 Testis-Enriched Genes via the CRISPR/Cas9 System Does Not Affect the Fertility of Male Mice. J. Reprod. Immunol..

[35] Bellil H., Ghieh F., Hermel E., Mandon-Pepin B., Vialard F. (2021). Human Testis-Expressed (TEX) Genes: A Review Focused on Spermatogenesis and Male Fertility. Basic Clin. Androl..

[36] Bruggeman J.W., Koster J., Lodder P., Repping S., Hamer G. (2018). Massive Expression of Germ Cell-Specific Genes Is a Hallmark of Cancer and a Potential Target for Novel Treatment Development. Oncogene.

[37] Erenpreisa J., Vainshelbaum N.M., Lazovska M., Karklins R., Salmina K., Zayakin P., Rumnieks F., Inashkina I., Pjanova D., Erenpreiss J. (2023). The Price of Human Evolution: Cancer-Testis Antigens, the Decline in Male Fertility and the Increase in Cancer. Int. J. Mol. Sci..

[38] da Silva V.L., Fonseca A.F., Fonseca M., da Silva T.E., Coelho A.C., Kroll J.E., de Souza J.E.S., Stransky B., de Souza G.A., de Souza S.J. (2017). Genome-Wide Identification of Cancer/Testis Genes and Their Association with Prognosis in a Pan-Cancer Analysis. Oncotarget.

[39] Kimmins S., Kotaja N., Davidson I., Sassone-Corsi P. (2004). Testis-Specific Transcription Mechanisms Promoting Male Germ-Cell Differentiation. Reproduction.

[40] Brown J.C. (2019). Control of Human Testis-Specific Gene Expression. PLoS ONE.

[41] Butsch T.J., Dubuisson O., Johnson A.E., Bohnert K.A. (2023). VCP Promotes tTAF-Target Gene Expression and Spermatocyte Differentiation by Downregulating Mono-Ubiquitylated H2A. Development.

[42] Jiang M., Gao Z., Wang J., Nurminsky D.I. (2018). Evidence for a Hierarchical Transcriptional Circuit in *Drosophila* Male Germline Involving Testis-Specific TAF and Two Gene-Specific Transcription Factors, Mod and Acj6. FEBS Lett..

[43] Mahadevaraju S., Fear J.M., Akeju M., Galletta B.J., Pinheiro M.M.L.S., Avelino C.C., Cabral-de-Mello D.C., Conlon K., Dell’Orso S., Demere Z. (2021). Dynamic Sex Chromosome Expression in Drosophila Male Germ Cells. Nat. Commun..

[44] Yang J.H., Hansen A.S. (2024). Enhancer Selectivity in Space and Time: From Enhancer–Promoter Interactions to Promoter Activation. Nat. Rev. Mol. Cell Biol..

[45] Sloutskin A., Shir-Shapira H., Freiman R.N., Juven-Gershon T. (2021). The Core Promoter Is a Regulatory Hub for Developmental Gene Expression. Front. Cell Dev. Biol..

[46] Danino Y.M., Even D., Ideses D., Juven-Gershon T. (2015). The Core Promoter: At the Heart of Gene Expression. Biochim. Biophys. Acta (BBA)—Gene Regul. Mech..

[47] Haberle V., Stark A. (2018). Eukaryotic Core Promoters and the Functional Basis of Transcription Initiation. Nat. Rev. Mol. Cell Biol..

[48] Cramer P. (2019). Organization and Regulation of Gene Transcription. Nature.

[49] Michiels F., Gasch A., Kaltschmidt B., Renkawitz-Pohl R. (1989). A 14 Bp Promoter Element Directs the Testis Specificity of the Drosophila beta 2 Tubulin Gene. EMBO J..

[50] Tung K.-F., Pan C.-Y., Chen C.-H., Lin W. (2020). Top-Ranked Expressed Gene Transcripts of Human Protein-Coding Genes Investigated with GTEx Dataset. Sci. Rep..

[51] Larkin A., Marygold S.J., Antonazzo G., Attrill H., dos Santos G., Garapati P.V., Goodman J.L., Gramates L.S., Millburn G., Strelets V.B. (2021). FlyBase: Updates to the *Drosophila melanogaster* Knowledge Base. Nucleic Acids Res..

[52] Gonzàlez-Porta M., Frankish A., Rung J., Harrow J., Brazma A. (2013). Transcriptome Analysis of Human Tissues and Cell Lines Reveals One Dominant Transcript per Gene. Genome Biol..

[53] Cummings B.B., Karczewski K.J., Kosmicki J.A., Seaby E.G., Watts N.A., Singer-Berk M., Mudge J.M., Karjalainen J., Satterstrom F.K., O’Donnell-Luria A.H. (2020). Transcript Expression-Aware Annotation Improves Rare Variant Interpretation. Nature.

[54] Ryu J.Y., Kim H.U., Lee S.Y. (2015). Human Genes with a Greater Number of Transcript Variants Tend to Show Biological Features of Housekeeping and Essential Genes. Mol. BioSyst..

[55] Ule J., Blencowe B.J. (2019). Alternative Splicing Regulatory Networks: Functions, Mechanisms, and Evolution. Mol. Cell.

[56] Baralle F.E., Giudice J. (2017). Alternative Splicing as a Regulator of Development and Tissue Identity. Nat. Rev. Mol. Cell Biol..

[57] Zhang Y., Qian J., Gu C., Yang Y. (2021). Alternative Splicing and Cancer: A Systematic Review. Signal Transduct. Target. Ther..

[58] Vedelek V., Kovács A.L., Juhász G., Alzyoud E., Sinka R. (2021). The Tumor Suppressor Archipelago E3 Ligase Is Required for Spermatid Differentiation in Drosophila Testis. Sci. Rep..

[59] Mortimer N.T., Moberg K.H. (2013). The Archipelago Ubiquitin Ligase Subunit Acts in Target Tissue to Restrict Tracheal Terminal Cell Branching and Hypoxic-Induced Gene Expression. PLoS Genet..

[60] Arama E., Bader M., Rieckhof G.E., Steller H. (2007). A Ubiquitin Ligase Complex Regulates Caspase Activation During Sperm Differentiation in Drosophila. PLoS Biol..

[61] Chen J.V., Buchwalter R.A., Kao L.-R., Megraw T.L. (2017). A Splice Variant of Centrosomin Converts Mitochondria to Microtubule-Organizing Centers. Curr. Biol..

[62] Vedelek V., Bodai L., Grézal G., Kovács B., Boros I.M., Laurinyecz B., Sinka R. (2018). Analysis of Drosophila Melanogaster Testis Transcriptome. BMC Genom..

[63] Uhlén M., Fagerberg L., Hallström B.M., Lindskog C., Oksvold P., Mardinoglu A., Sivertsson Å., Kampf C., Sjöstedt E., Asplund A. (2015). Tissue-Based Map of the Human Proteome. Science.

[64] Li J.J., Huang H., Bickel P.J., Brenner S.E. (2014). Comparison of *D. Melanogaster* and *C. Elegans* Developmental Stages, Tissues, and Cells by modENCODE RNA-Seq Data. Genome Res..

[65] Jurisch-Yaksi N., Wachten D., Gopalakrishnan J. (2024). The Neuronal Cilium—A Highly Diverse and Dynamic Organelle Involved in Sensory Detection and Neuromodulation. Trends Neurosci..

[66] FitzGerald P.C., Sturgill D., Shyakhtenko A., Oliver B., Vinson C. (2006). Comparative Genomics of Drosophila and Human Core Promoters. Genome Biol..

[67] Green P., Ewing B., Miller W., Thomas P.J., Green E.D. (2003). NISC Comparative Sequencing Program Transcription-Associated Mutational Asymmetry in Mammalian Evolution. Nat. Genet..

[68] Vinckenbosch N., Dupanloup I., Kaessmann H. (2006). Evolutionary Fate of Retroposed Gene Copies in the Human Genome. Proc. Natl. Acad. Sci. USA.

[69] Marques A.C., Dupanloup I., Vinckenbosch N., Reymond A., Kaessmann H. (2005). Emergence of Young Human Genes after a Burst of Retroposition in Primates. PLoS Biol..

[70] Dorus S., Freeman Z.N., Parker E.R., Heath B.D., Karr T.L. (2008). Recent Origins of Sperm Genes in Drosophila. Mol. Biol. Evol..

[71] Betrán E., Thornton K., Long M. (2002). Retroposed New Genes Out of the X in *Drosophila*. Genome Res..

[72] Su Q., He H., Zhou Q. (2021). On the Origin and Evolution of Drosophila New Genes during Spermatogenesis. Genes.

[73] Peng J., Zhao L. (2024). The Origin and Structural Evolution of de Novo Genes in Drosophila. Nat. Commun..

[74] Witt E., Benjamin S., Svetec N., Zhao L. (2019). Testis Single-Cell RNA-Seq Reveals the Dynamics of de Novo Gene Transcription and Germline Mutational Bias in Drosophila. eLife.

[75] Titus-McQuillan J.E., Nanni A.V., McIntyre L.M., Rogers R.L. (2023). Estimating Transcriptome Complexities across Eukaryotes. BMC Genom..

[76] Chen L., Bush S.J., Tovar-Corona J.M., Castillo-Morales A., Urrutia A.O. (2014). Correcting for Differential Transcript Coverage Reveals a Strong Relationship between Alternative Splicing and Organism Complexity. Mol. Biol. Evol..

[77] Suter B., Schnappauf G., Thoma F. (2000). Poly(dA.dT) Sequences Exist as Rigid DNA Structures in Nucleosome-Free Yeast Promoters in Vivo. Nucleic Acids Res..

[78] Travers A., Muskhelishvili G. (2015). DNA Structure and Function. FEBS J..

[79] Harteis S., Schneider S. (2014). Making the Bend: DNA Tertiary Structure and Protein-DNA Interactions. Int. J. Mol. Sci..

[80] Naro C., Cesari E., Sette C. (2021). Splicing Regulation in Brain and Testis: Common Themes for Highly Specialized Organs. Cell Cycle.

[81] Lee J.E., Gleeson J.G. (2011). Cilia in the Nervous System: Linking Cilia Function and Neurodevelopmental Disorders. Curr. Opin. Neurol..

[82] Trinh I., Boulianne G.L. (2013). Modeling Obesity and Its Associated Disorders in *Drosophila*. Physiology.

[83] Musselman L.P., Kühnlein R.P. (2018). *Drosophila* as a Model to Study Obesity and Metabolic Disease. J. Exp. Biol..

[84] Alfa R.W., Kim S.K. (2016). Using *Drosophila* to Discover Mechanisms Underlying Type 2 Diabetes. Dis. Models Mech..

[85] Brattig-Correia R., Almeida J.M., Wyrwoll M.J., Julca I., Sobral D., Misra C.S., Di Persio S., Guilgur L.G., Schuppe H.-C., Silva N. (2024). The Conserved Genetic Program of Male Germ Cells Uncovers Ancient Regulators of Human Spermatogenesis. eLife.

[86] Leader D.P., Krause S.A., Pandit A., Davies S.A., Dow J.A.T. (2018). FlyAtlas 2: A New Version of the Drosophila Melanogaster Expression Atlas with RNA-Seq, miRNA-Seq and Sex-Specific Data. Nucleic Acids Res..

[87] Hu Y., Flockhart I., Vinayagam A., Bergwitz C., Berger B., Perrimon N., Mohr S.E. (2011). An Integrative Approach to Ortholog Prediction for Disease-Focused and Other Functional Studies. BMC Bioinform..

[88] Kuznetsov D., Tegenfeldt F., Manni M., Seppey M., Berkeley M., Kriventseva E.V., Zdobnov E.M. (2023). OrthoDB V11: Annotation of Orthologs in the Widest Sampling of Organismal Diversity. Nucleic Acids Res..

[89] Garcia J., Korhonen T. DIOPT: Extremely Fast Classification Using Lookups and Optimal Feature Discretization. Proceedings of the 2020 International Joint Conference on Neural Networks (IJCNN).

[90] Lindskog C. (2016). The Human Protein Atlas—An Important Resource for Basic and Clinical Research. Expert Rev. Proteom..

[91] Thomas P.D., Ebert D., Muruganujan A., Mushayahama T., Albou L.-P., Mi H. (2022). PANTHER: Making Genome-Scale Phylogenetics Accessible to All. Protein Sci..

[92] Mi H., Muruganujan A., Casagrande J.T., Thomas P.D. (2013). Large-Scale Gene Function Analysis with the PANTHER Classification System. Nat. Protoc..

[93] Mi H. (2004). The PANTHER Database of Protein Families, Subfamilies, Functions and Pathways. Nucleic Acids Res..

[94] Shannon P., Markiel A., Ozier O., Baliga N.S., Wang J.T., Ramage D., Amin N., Schwikowski B., Ideker T. (2003). Cytoscape: A Software Environment for Integrated Models of Biomolecular Interaction Networks. Genome Res..

[95] Su G., Morris J.H., Demchak B., Bader G.D. (2014). Biological Network Exploration with Cytoscape 3. Curr. Protoc. Bioinform..

[96] AltschuP S.F., Gish W., Miller W., Myers E.W., Lipman D.J. (1990). Basic Local Alignment Search Tool. J. Mol. Biol..

[97] Bailey T.L., Johnson J., Grant C.E., Noble W.S. (2015). The MEME Suite. Nucleic Acids Res..

